# **Medial temporal lobe atrophy ratings in a large 75-year-old population-based cohort: gender-corrected and education-corrected normative data**

**DOI:** 10.1007/s00330-017-5103-6

**Published:** 2017-11-09

**Authors:** V. Velickaite, D. Ferreira, L. Cavallin, L. Lind, H. Ahlström, L. Kilander, E. Westman, E.-M. Larsson

**Affiliations:** 1 Department of Surgical Sciences, Radiology, Uppsala University, SE-751 85 Uppsala, Sweden; 2 Department of Neurobiology, Care sciences and Society, Centre for Alzheimer’s Research, Division of Clinical Geriatrics, Karolinska Institutet, SE-104 35 Stockholm, Sweden; 3 Karolinska University Hospital in Huddinge and Department of Clinical Science, Intervention and Technology in Karolinska Institute, SE-141 86 Stockholm, Sweden; 4 Department of Public Health and Caring Sciences, Geriatrics, Uppsala University, SE-751 22 Uppsala, Sweden

**Keywords:** Medial temporal lobe atrophy (MTA), Scheltens’s scale, Dementia, Cognitive test, Longitudinal analysis, Population-based

## Abstract

**Objectives:**

To find cut-off values for different medial temporal lobe atrophy (MTA) measures (right, left, average, and highest), accounting for gender and education, investigate the association with cognitive performance, and to compare with decline of cognitive function over 5 years in a large population-based cohort.

**Methods:**

Three hundred and ninety 75-year-old individuals were examined with magnetic resonance imaging of the brain and cognitive testing. The Scheltens’s scale was used to assess visually MTA scores (0–4) in all subjects. Cognitive tests were repeated in 278 of them after 5 years. Normal MTA cut-off values were calculated based on the 10th percentile.

**Results:**

Most 75-year-old individuals had MTA score ≤2. Men had significantly higher MTA scores than women. Scores for left and average MTA were significantly higher in highly educated individuals. Abnormal MTA was associated with worse results in cognitive test and individuals with abnormal right MTA had faster cognitive decline.

**Conclusion:**

At age 75, gender and education are confounders for MTA grading. A score of ≥2 is abnormal for low-educated women and a score of ≥2.5 is abnormal for men and high-educated women. Subjects with abnormal right MTA, but normal MMSE scores had developed worse MMSE scores 5 years later.

***Key Points*:**

• *Gender and education are confounders for MTA grading.*

• *We suggest cut-off values for 75-year-olds, taking gender and education into account.*

• *Males have higher MTA scores than women.*

• *Higher MTA scores are associated with worse cognitive performance.*

## Introduction

Medial temporal lobe atrophy (MTA) is common in dementia and is also frequently seen in mild cognitive impairment (MCI). However, it can also be seen in normal aging [1, 2]. MTA has been shown to be a very strong predictor of the progression of MCI to Alzheimer’s disease (AD) [3, 4]. This finding is, therefore, helpful in the clinical radiological evaluation of patients with suspected dementia disorders.

In the 1984 NINCDS-ADRDA criteria for the diagnosis of probable AD, imaging was included among the supportive criteria [5]. Since then, important advances have occurred in the understanding of the pathophysiological processes involved in AD, and the ability to detect AD in early stages has improved. These criteria have been revised, and magnetic resonance imaging (MRI) evidence of MTA is included as a biomarker that increases the confidence of a clinical diagnosis of AD [5, 6]. However, it is of utmost importance to define the cut-off values that distinguish abnormal from normal for biomarkers like MTA to increase their use in clinical practice.

**Fig. 1.**
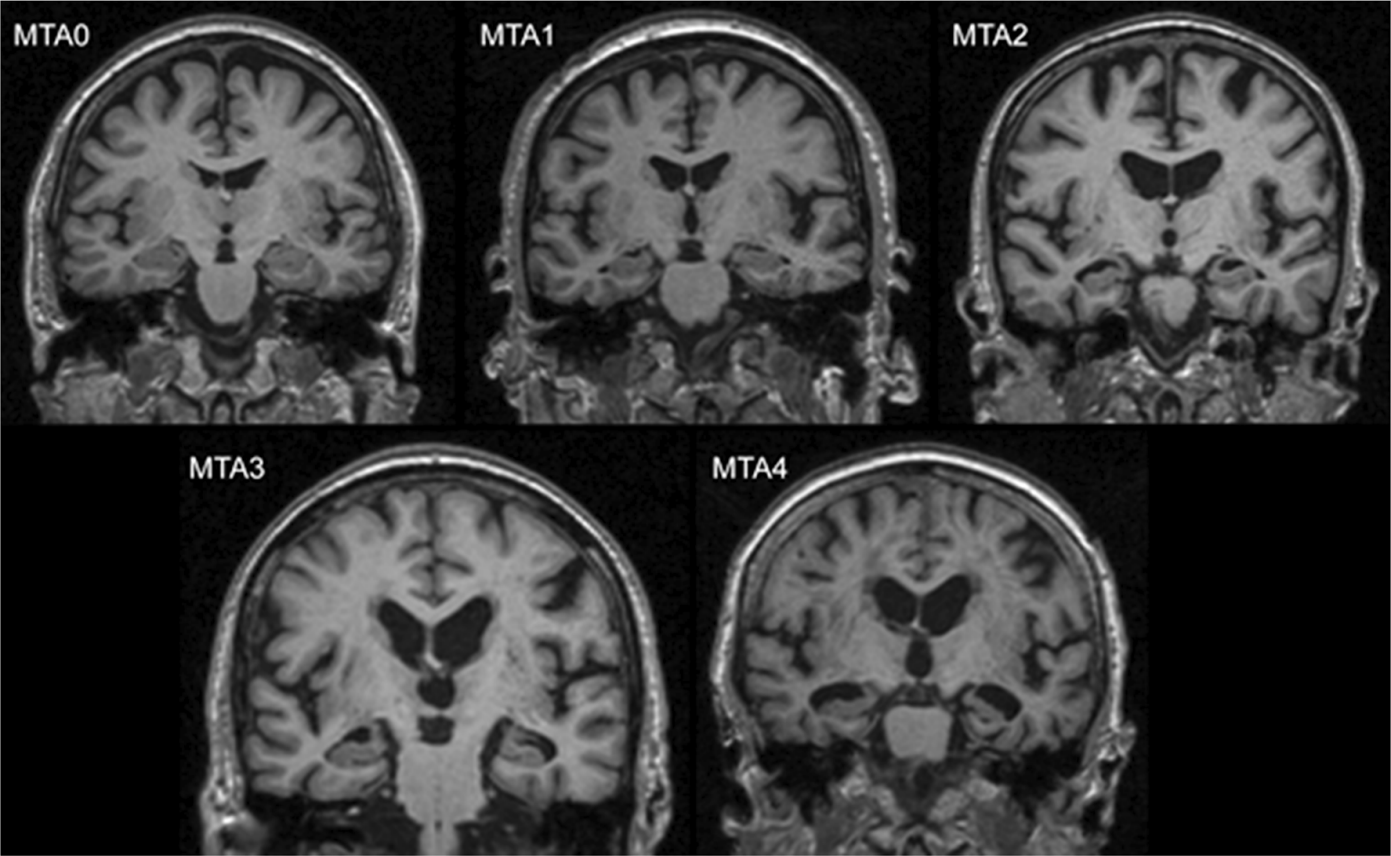
Medial temporal lobe atrophy (MTA) grades 0-4 with increasing severity (example images from our study). MTA = medial temporal lobe atrophy.

Volumetric methods for the evaluation of the hippocampus, although increasingly used, are still rarely applied in everyday clinical practice since they are complex and time consuming [7–9]. In 1992, Scheltens et al. published a rating scale for the visual assessment of MTA on MRI that is now the most commonly recommended tool to assess MTA [10]. The predictive accuracy of the Scheltens’s MTA scale to distinguish between AD and healthy individuals has been shown to be similar to that of computer-driven techniques [3, 9, 11]. The scale can also be used on computed tomography (CT) brain scans, with similar accuracy [12, 13]. This scale is based on the evaluation of coronal images of the brain and has five grades (0 to 4). The evaluation criteria comprise the width of the choroid fissure, the width of the temporal horn, and the height of the hippocampus [14]. Score 0 refers to no atrophy, score 1 shows widening of the chorioid fissure, score 2 includes additional widening of the temporal horn of the lateral ventricle and slightly decreased hippocampal height, score 3 includes moderate loss of hippocampal volume, and 4 shows end-stage increase of all these findings (Fig. 1). In the original study of a small group of patients with AD, 81 % had MTA scores from 2 to 4, while 67 % of a small control group had scores 0 or 1. None of the controls had score 4, and one AD patient had score 0 [12, 15].

Today, Scheltens’s scale is used for the visual assessment of MTA (MTA score) in both clinical practice and research as an imaging biomarker for the diagnosis of AD, but also for other types of neurodegenerative disorders [14, 16]. The MTA score is also frequently used as an outcome variable in clinical trials [17]. Several studies have evaluated MTA scores in different age groups, and different age-corrected cut-off values for abnormality have been proposed [2, 18–21]. A score of 0 or 1 is considered normal at all ages, and a score of 4 is regarded as abnormal at all ages. Barkhof et al. stated that an MTA score of 1 can be regarded as normal in patients younger than 75 years, and an MTA score of ≤2 can be considered normal in individuals older than 75 years [14]. Later, Cavallin et al. found that normal values for non-demented 70- to 80-year-olds are MTA ≤ 2 and MTA ≤ 3 for those older than 80 years. Thus, the age below which MTA 2 is considered abnormal may be either 75 or 70 years. Hence, there is some controversy regarding different cut-off values for atrophy scores, especially around age 75 years [14]. Notably, earlier studies were based mainly on highly selected samples or clinical settings, and it is important to also investigate these cut-offs in population-based samples.

The hippocampus is often larger on the right than on the left side in normal individuals, and the MTA score can also be asymmetric, with a higher score on one side [2, 22, 23]. Some studies have used the highest score on one side as the deciding factor [12, 20, 24]. Other studies have, however, calculated the average between left and right MTA scores and found that an average MTA score above 1.5 can be considered abnormal in persons 75 years and older [20, 21, 24].

The aim of this study was to evaluate MTA using Scheltens’s scale and to determine the association between MTA scores and cognitive performance, gender, and education. Since 75 years has previously been proposed as a critical age for MTA, we sought to define normal MTA scores in a large homogeneous 75-year-old population. This has, to our knowledge, not been done previously. In addition, we compared the used MTA measures (right, left, average, and highest score) in the same cohort, which has also not been addressed before. New, accurate cut-off values that can be implemented in clinical practice are needed. Finally, since the prognostic value of MTA scoring is of clinical interest, we evaluated progression over a 5-year course.

## Material and methods

### Subjects

The Prospective Investigation of the Vasculature in Uppsala Seniors (PIVUS) is a population-based study of 1016 individuals recruited at age 70 years [25, 26]. The subjects were included randomly from the population register of the municipality and previous or current diseases were not exclusion criteria. They reported any history of diseases, such as myocardial infarct (MI), stroke, angina pectoris (AP), congestive heart failure, diabetes, and previous coronary artery bypass/percutaneous coronary angioplasty (CABG/PTCA). Further, they reported history of used medications, such as any antihypertensive or cardiovascular medication, diuretics, statins, oral antidiabetics drugs, and others. MRI of the brain was undertaken in 406 of these subjects (randomly selected) 5 years later, at age 75 years.

Cognitive evaluation of individuals at age 75 years was done with the Mini-Mental State Examination (MMSE), the 7 Minute Screening Battery, including Benton temporal orientation (BTO), enhanced cued free recall (ECFR) of 16 items, clock drawing test and semantic verbal fluency (animals per 1 min), and the Trail Making Test A and B (TMT-A and TMT-B) [27–29]. None of the individuals had a diagnosis of dementia at the time of inclusion.

Subjects were stratified into two groups based on the level of education (up to 8 years of education was regarded as “low education” and 9 years or more as “high education”). None of the individuals had a diagnosis of dementia and only one individual, female with “high education level”, had mild cognitive impairment- at the time of inclusion in our study at the age of 75 years. Only subjects with an available brain MRI and information about educational level (390 individuals) were included in this study. Descriptive data about subjects is presented in Table 1.

**Table 1.** Descriptive data of the study cohort.

Gender, women/men (%) Education, low/high (%) Comorbidities (%) Medication (%) Dementia diagnosis at baseline, N MMSE, mean ± SD	47/53 57/43 85 80 0 29±1
7 Minute test:	
BTO, mean ± SD	0.68±3.34
ECRT-free recall, mean ± SD	9.70±2.49
ECRT-cued recall, mean ± SD	6.00±2.28
Clock drawing, mean ± SD	6.5±1.10
Semantic fluency, mean ± SD	20.40±6.61
TMT-A, mean ± SD	55±21.83
TMT-B, mean ± SD	149±117.47

SD= Standard deviation. MMSE = mini-mental state examination; BTO = Benton temporal orientation; ECRT = Enhanced cued recall task; TMT = trail making test.

A follow-up cognitive examination with MMSE was performed at age 80 years in 278 of the individuals. The local ethics committee approved the study, and all individuals provided written informed consent.

### MRI and visual ratings of MTA

MRI of the brain was performed with a 1.5 T MR scanner (Intera, Philips Healthcare, Best, the Netherlands). A sagittal T1-weighted 3D gradient echo sequence (echo time 4.0 ms, repetition time 8.6 ms, flip angle 8 °, resolution 0.94x0.94x1.2 mm and matrix 256x256x170) was interactively reconstructed to 1.2 mm thick coronal images in a Picture Archiving and Communication System (VuePACS, Carestream; Carestream Health, Inc., Rochester, NY, USA) for visual assessment. The coronal reconstructed images were angled parallel to the posterior contour of the brainstem.

For MTA rating, coronal images located just posterior to the amygdala in the area of the cerebral peduncles were used. One operator (VV) using Scheltens’s scale performed the visual rating. Randomly selected individuals (*n* = 50) were rated by a second operator (LC), and interrater reliability was calculated. Also, randomly selected individuals (n = 20) were rated again by the first operator (VV) and intrarater reliability was calculated. Four scores were investigated in the current study: right and left sides MTA separately, average MTA and MTA highest (highest out of left and right score when they were different).

### Statistics

Kendall rank and Spearman’s rank correlations were used to investigate associations between variables. Mann-Whitney U and Kruskal-Wallis tests were used for group comparisons, and one-way ANCOVA was used for group comparisons while accounting for the effect of gender and education. *P*-values in all these analyses (including post hoc paired tests) were adjusted with the Benjamini-Hochberg correction for multiple comparisons. The chi-squared test was used for categorical variables. Multiple linear regression and ordinal regression were used to investigate the effect of gender and education, and their interaction on the different MTA measures. Mixed ANCOVA was used to test the interaction between a within-subjects factor and a between-subjects factor while accounting for the effect of gender and education. Inter-rater and intra-rater reliability were assessed with the weighted Kappa test, and results were interpreted following the criteria of Landis and Koch (j < 0, no agreement; j = 0–0.20, slight agreement; j = 0.21–0.40, fair agreement; j < 0.41–0.60, moderate agreement; j = 0.61–0.80, substantial agreement; j = 0.81–1.0, almost perfect agreement [30]. All the results were considered significant when *P*≤0.05 (two-tailed).

## Results

**Fig. 2.**
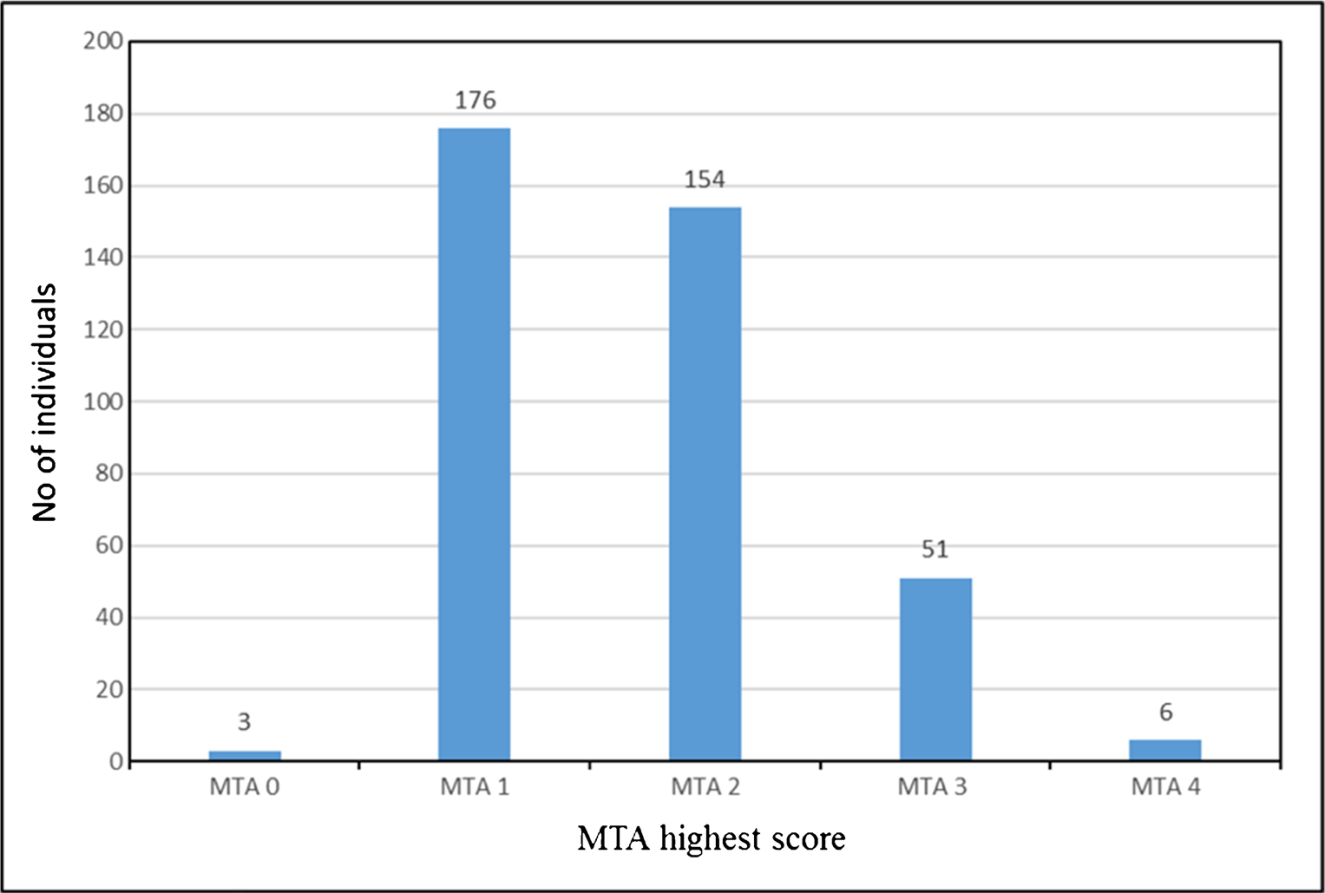
Distribution of the MTA highest scores. No = number, MTA = medial temporal lobe atrophy.

Among the 390 individuals, all 75 years old, the gender distribution was balanced (47 % female; X^2^_(1)_ = 1.026; *P* = 0.311). There were significantly more participants with low education (57 % had less than 8 years of education, 43 % had 9 or more years of education; X^2^_(1)_ = 8.626; *P* = 0.003). MMSE scores ranged from 21 to 30 (median 29). Inter-rater reliability for visual MTA rating showed substantial agreement (weighted Kappa was 0.62 and 0.67 for the right and the left side, respectively). Intra-rater reliability showed substantial to almost perfect agreement (weighted Kappa was 0.84 and 0.79 for the right and the left side, respectively). The distribution of MTA highest scores across our cohort is shown in Fig 2.

### Influence of gender and education on the MTA measures

**Fig. 3.**
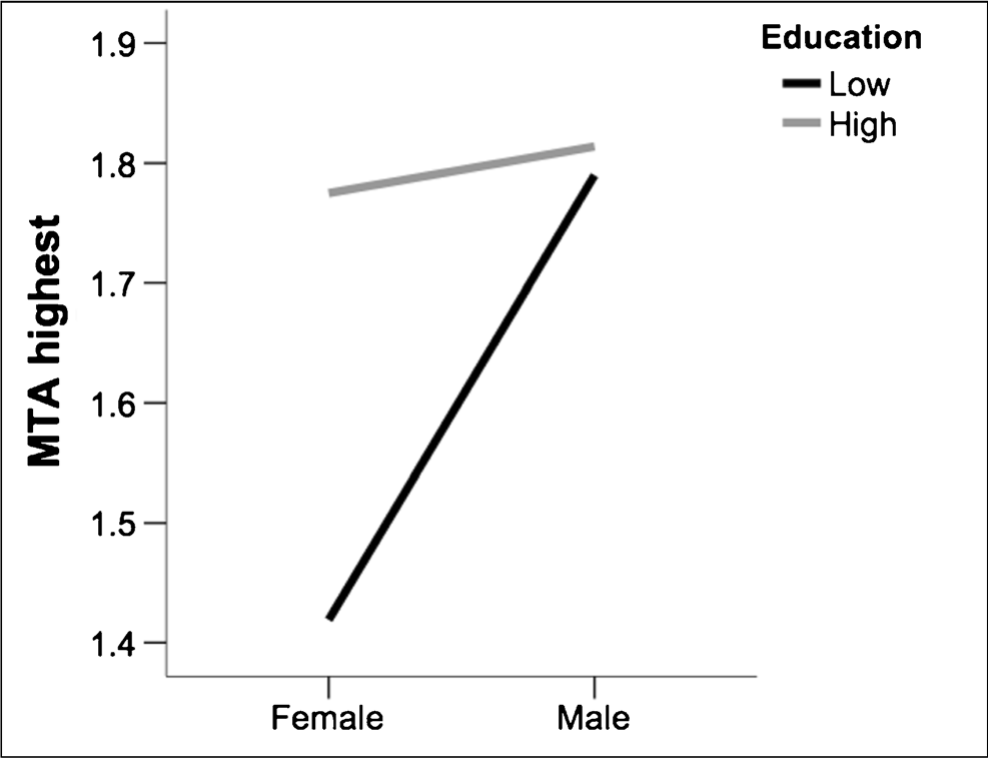
Interaction between gender and education on MTA highest. Education is coded as low “<9 years of education” and high “≥9 years of education”. MTA = medial temporal atrophy. MTA = medial temporal atrophy.

Table 2 shows that gender had a significant effect on the four MTA measures, with males having significantly higher scores than females. Scores for left MTA and average MTA were also significantly higher in highly educated individuals. The interaction between gender and education indicated that scores for MTA highest were lower in females, but only if they had low education (Fig. 3).

**Table 2.** Effect of gender and education on MTA measures

MTA measure	Model	Predictor	Estimate (*) / ß	*P*-value
MTA right	R^2^=2%; X^2^_(2)_=5.982; *P*=0.050	Gender	-0.410 *	0.041
MTA left	R^2^=6%; X^2^_(2)_=20.962; *P*<0.001	Gender	-0.746 *	<0.001
		Education	-0.574 *	0.005
MTA average	R^2^=3%; F_(2, 387)_=6.470; *P*=0.002	Gender	0.148	0.003
		Education	0.104	0.039
MTA highest	R^2^=6%; X^2^_(3)_=21.148; *P*<0.001	Gender x Education	-0.842 *	0.032

Gender is coded as 0 “female” and 1 “male”. Education is coded as 0 “<9 years of education” and 1 “≥9 years of education”. * Ordinal regression: male and high education are the reference categories; thus, negative estimates mean lower MTA scores in women and in low educated individuals. R^2^ was estimated with the Nagelkerke’s function in ordinal regression. MTA = medial temporal atrophy.

### Normative data and cut-offs for MTA measures by gender and education

Based on the above-mentioned results, the sample was stratified into four groups: female + low education (*n* = 105); female + high education (*n* = 80); male + low education (*n* = 119); and male + high education (*n* = 86). Normative data on the different MTA measures for these four groups are displayed in Table 3. The Kruskal-Wallis test confirmed the effect of gender and education on the MTA measures. Derived 10th percentile cut-off scores indicated that values between 2 and 3 (and higher) were abnormal, depending on the MTA measure, gender and education (Table 3). By applying these cut-offs, MTA right was abnormal in 64 individuals (16 %), MTA left in 83 individuals (21 %), MTA average in 69 individuals (18 %), and MTA highest in 88 individuals (23 %).

**Table 3.** Normative data and cut-off values for MTA measures in relation to gender and education.

	Female Low Education (N = 105)	Female High Education (N = 80)	Male Low Education (N = 119)	Male High Education (N = 86)	Kruskal-Wallis
MTA right					
Mean	1.35	1.53	1.56	1.56	X^2^_(3)_=6.838; *P*=0.077
Median	1.00	1.00	1.00	1.50	
SD	0.60	0.73	0.72	0.71	
Range	0–4	0–3	0–4	0–3	
Percentile 10th (cut-off)	2	3	3	3	
MTA left					
Mean	1.26	1.50	1.56 ^a^	1.69 ^a^	X^2^_(3)_=20.981; *P*=0.003
Median	1.00	1.00	1.00	2.00	
SD	0.56	0.73	0.81	0.76	
Range	0–4	1–4	0–4	0–3	
Percentile 10th (cut-off)	2	2	3	3	
MTA average					
Mean	1.31	1.51 ^a^	1.56 ^a^	1.62 ^a^	X^2^_(3)_=17.180; *P*=0.020
Median	1.00	1.50	1.50	1.50	
SD	0.53	0.63	0.69	0.67	
Range	0–4	0.5 –3.5	0–4	0–3	
Percentile 10th (cut-off)	2	2.5	2.5	2.5	
MTA highest					
Mean	1.42	1.78 ^a^	1.79 ^a^	1.81 ^a^	X^2^_(3)_=20.499; *P*=0.003
Median	1.00	2.00	2.00	2.00	
SD	0.65	0.76	0.80	0.76	
Range	0–4	1–4	0–4	0–3	
Percentile 10th (cut-off)	2	3	3	3	

Education is coded as low when <9 years of education and high when ≥9 years of education. All *P*-values are adjusted with the Benjamini-Hochberg correction for multiple comparisons. ^a^ = Significantly different from Female Low Education. MTA = medial temporal atrophy; SD = standard deviation.

The “Percentile 10th (cut-off) value” (and higher values) is regarded as abnormal in each group.

In 283 individuals (73 %), scores were normal for all MTA measures, and in 40 individuals (10 %) scores were abnormal for all four MTA measures. Thus, findings were consistent in 323 individuals. Findings were inconsistent in 67 individuals. In 19 individuals (5 %) only the MTA left score was abnormal, in 19 individuals (5 %) scores in two MTA measures were abnormal, and in 29 individuals (7 %) scores in three MTA measures were abnormal. MTA highest detected all the consistent and inconsistent cases except for the 19 individuals detected only by MTA left.

### Clinical associations between MTA grade and cognitive tests at baseline and after 5 years

Individuals with abnormal MTA average had worse performance in the MMSE, memory, and TMT-B (Table 4). TMT-B performance was also reduced in individuals with abnormal MTA highest.

**Table 4.** Association between MTA cut-off values and cognitive tests at age 75 years.

	MTA right cut-off	MTA left cut-off	MTA average cut-off	MTA highest cut-off
MMSE	0.497	0.452	0.024*	0.215
BTO	0.084	0.452	0.088	0.077
ECRT-free recall	0.497	0.272	0.014*	0.312
ECRT-cued recall	0.497	0.452	0.065	0.359
Clock drawing	0.497	0.452	0.414	0.359
Semantic fluency	0.312	0.452	0.216	0.126
TMT-A	0.497	0.597	0.410	0.359
TMT-B	0.056	0.329	0.002*	0.032*

Values in the table represent the *P*-values from the comparison between MTA normal and MTA abnormal. * *P*<0.05 (to facilitate reading). All *P*-values are adjusted with the Benjamini-Hochberg correction for multiple comparisons. MTA = medial temporal atrophy; MMSE = mini-mental state examination; BTO = Benton temporal orientation; ECRT = Enhanced cued recall task; TMT = trail making test.

**Fig. 4.**
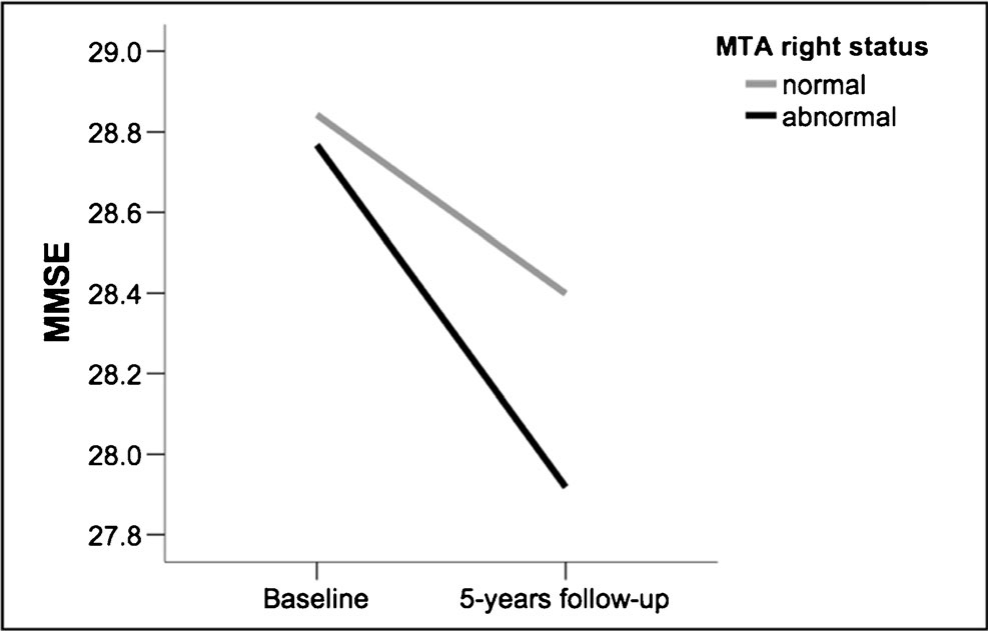
Changes in MMSE performance after 5 years related to MTA right. Values in the y-axis are the estimated means for MMSE from the mixed ANCOVA when including gender and education as covariates. MTA = medial temporal atrophy; MMSE = mini-mental state examination.

Mixed ANCOVA showed that there was a trend towards an interaction between time (baseline vs. 5 years’ follow-up) and MTA status (normal vs*.* abnormal) only for the MTA right measure (F _(1, 274)_ = 3.835; *P* = 0.051), suggesting faster cognitive decline, measured by the MMSE, in the group with abnormal MTA right at baseline (Fig. 4). No significant interactions between time and MTA status were found for MTA left, MTA average, and MTA highest.

## Discussion

The evaluation of atrophy of the medial temporal lobes in elderly patients is important in the diagnostic work-up of clinical dementia. Nevertheless, even if an individual has an abnormal MTA score, it should always be remembered that the MTA score alone cannot be the deciding factor in establishing a diagnosis of a dementia disorder.

In this study, we investigated the distribution of MTA scores in a homogeneous 75-year-old population-based cohort and the association between MTA scores and cognitive performance, gender and education. All participants were recruited from a population in a geographically well-defined region and were closely followed. Subjects self-reported various diseases, commonly found at this age, as well as ongoing medication, which were not exclusion criteria, as it would be very difficult to find persons at older age without any medical problems. The cohort was homogeneous with regard to age, all were 75 years old, and gender distribution.

The present study shows that gender and education are important confounders when interpreting MTA scores and deriving clinical cut-offs at the age of 75 years. Gender is the main confounder, men having higher MTA scores than women. This is in line with previous studies that had shown that the volume of the hippocampus is smaller in healthy men than in women [31, 32].

Education is another confounder, although not as important as gender. Individuals with higher education have better cognitive tests results, even though they have higher MTA scores. This is supported by the theory regarding the cognitive reserve of the brain, which states that inter-individual differences in the effective usage of brain networks or cognitive processing strategies is relevant for preserving better cognition despite pathological processes. Previous studies have suggested that education compensates for the impact of MTA on cognition: individuals with less than 8 years of education have two times higher risk of developing dementia and in well-educated individuals the effect of MTA on cognition is weaker than in less-educated subjects [33–35].

Subjects with normal MMSE scores and abnormal grade of MTA at age 75 years had developed worse MMSE scores 5 years later. This is in line with previous studies based on patients with known cognitive impairment [3, 36, 37]. As mentioned above, in higher educated individuals, cognitive reserve may to some degree compensate for more severe MTA, but when cognitive impairment emerges, the decline is more rapid. This has also been shown in other studies with normal populations [34].

Our results showed a slight asymmetry of atrophy scores on the right and left sides, with more cases with abnormal MTA on the left than on the right side. One possible explanation could be that the left hippocampus is more vulnerable because of a smaller premorbid size. Previous studies have shown that asymmetry with smaller hippocampus on the left side is common in normal individuals without dementia, and also in term and preterm newborns [23, 38, 39].

Our findings suggested faster cognitive decline in the group with abnormal MTA right at baseline, which is in line with a previous study showing that higher MTA on the right side predicted conversion of MCI to dementia [40]. However, another study suggested that left-sided hippocampal atrophy is potentially a better neuroradiological biomarker for cognitive decline [41]. Thus, results may differ between studies, possibly depending on different study designs and insufficient number of subjects.

We found a cut-off score of ≥2 for abnormal MTA in low-educated women, but for men and for high-educated women, the cut-off score was ≥2.5 (average of left and right) or ≥3 (highest of left and right) (Table 3). This is partly in agreement with previous studies showing that a MTA score of >2 can be regarded as abnormal in individuals older than 70 to 75 years [2, 14]. It should be emphasised that cut-off values differ depending on gender and educational level. To our knowledge, this has not been addressed previously. A recent study reported that an optimal cut-off value for age 75 years is ≥2 [42]. However, that study was based on patients with AD and subjective cognitive impairment from a memory clinic, which was a different study population than our community-based cohort.

Strengths of our study are that it is population-based, all subjects have the same age and the gender distribution is almost symmetrical. Thus, MTA grade and its correlation with education and gender are not affected by age. In addition, we have 5 years’ follow-up of the MMSE. We have provided data not only for left and right MTA scores, but also for the average and the highest score, since they are all used clinically, but the type of cut-off values is not evident in the literature. In addition, we appear to be the first group to provide gender- and education-corrected cut-off values.

A limitation of this study is that we did not determine cut-off values and investigated diagnostic performance in a group of patients with clinical dementia. To circumvent this, our definition of abnormality was based on the 10th percentile, which is a common and also valid procedure in studies of this kind [43]. Complementarily, we demonstrated the prognostic value of right MTA for cognitive decline, although the clinical value of the other MTA measures remains questionable. Also, clinical usefulness of the right MTA score for prediction of longitudinal cognitive decline could not be calculated because only 7 individuals developed cognitive impairment (MMSE<24) at follow-up. However, this further supports the fact that these cut-offs have been derived from a rather healthy and cognitively stable sample.

In conclusion, most of the 75-year-old individuals without recognised cognitive impairment in this study had a MTA score of ≤2. A significant confounding factor in this study was gender, followed by education. We found different abnormal cut-off scores depending on gender and education level: for low-educated women, the cut-off score was ≥2, but for men and high-educated women, a score of ≥2.5 (average) or ≥3 (highest) is considered abnormal.
